# The salutary effects of diphenyldifluoroketone EF24 in liver of a rat hemorrhagic shock model

**DOI:** 10.1186/s13049-015-0098-y

**Published:** 2015-02-03

**Authors:** Vivek R Yadav, Alamdar Hussain, Jun Xie, Stanley Kosanke, Vibhudutta Awasthi

**Affiliations:** Department of Pharmaceutical Sciences, University of Oklahoma Health Science Center, 1110 North Stonewall Avenue, Oklahoma City, OK 73117 USA; Department of Comparative Medicine, University of Oklahoma Health Science Center, 1110 North Stonewall Avenue, Oklahoma City, OK USA

**Keywords:** EF24, Liver injury, Hemorrhagic shock, Resuscitation, Necrosis

## Abstract

**Background:**

Liver is a target for injury in low flow states and it plays a central role in the progression of systemic failure associated with hemorrhagic shock. Pharmacologic support can help recover liver function even after it has suffered extensive damage during ischemia and reperfusion phases. In this work we assessed the efficacy of a diphenyldifluoroketone EF24, an IKKβ inhibitor, in controlling hepatic inflammatory signaling caused by hemorrhagic shock in a rat model.

**Methods:**

Sprague Dawley rats were bled to about 50% of blood volume. The hemorrhaged rats were treated with vehicle control or EF24 (0.4 mg/kg) after 1 h of hemorrhage without any accompanying resuscitation. The study was terminated after additional 5 h to excise liver tissue for biochemical analyses and histology.

**Results:**

EF24 treatment alleviated hemorrhagic shock-induced histologic injury in the liver and restored serum transaminases to normal levels. Hemorrhagic shock induced the circulating levels of CD163 (a marker for macrophage activation) and CINC (an IL-8 analog), as well as myeloperoxidase activity in liver tissue. These markers of inflammatory injury were reduced by EF24 treatment. EF24 treatment also suppressed the expression of the Toll-like receptor 4, phospho-p65/Rel A, and cyclooxygenase-2 in liver tissues, indicating that it suppressed inflammatory pathway. Moreover, it reduced the hemorrhagic shock-induced increase in the expression of high mobility group box-1 protein. The evidence for apoptosis after hemorrhagic shock was inconclusive.

**Conclusion:**

Even in the absence of volume support, EF24 treatment suppresses pro-inflammatory signaling in liver tissue and improves liver functional markers in hemorrhagic shock.

## Background

The morbidity and mortality associated with hemorrhagic shock (HS) is a composite result of systemic inflammatory response syndrome (SIRS) caused by hemorrhage and reperfusion. Liver is one of the vital organs in this coordinated bodily response to HS and approximately 20% of HS victims exhibit some degree of liver dysfunction [1,2]. Given the role played by liver in metabolic, excretory, and homeostatic mechanisms, liver injury is directly linked to the systemic deterioration which may nullify the effectiveness of resuscitation strategies in shock. At the same time, liver is sufficiently resilient to recover even after it has suffered extensive damage, suggesting that effective pharmacologic strategies early in shock may help liver tissue survive ischemic and reperfusion injury. Several pharmacologic substances such as 2-mercaptopropionyl glycine [3], melatonin [4], and ethyl pyruvate [5] are being investigated in preclinical models to maintain liver function after HS and reperfusion injury. It is notable that in a multifaceted pathology of shock, mono-therapies, such as infliximab against tumor necrosis factor-alpha (TNF-α) or Xigris™ targeting activated protein C, fail to provide expected clinical outcomes. Therefore, compounds with pleiotropic effects via modulation of transcriptional factors may be more effective because of their influence on multiple pathways involved in SIRS pathogenesis. The molecular pathways influenced by transcription factor nuclear factor kB (NF-kB) play the most important role in orchestrating the inflammatory response through the cytokines and the transcriptional control.

The activation of inflammatory pathways, mediated by induction of inducible nitric oxide synthase (iNOS) and cyclooxygenase-2 (COX-2) genes, is believed to contribute to the liver injury in shock [6]. Although iNOS and COX-2 pathways can also be controlled by transcription elements hypoxia-inducing factor-1, activator protein-1, and p53 [7], NF-κB is the most ubiquitous rapid response transcription factor involved in these inflammatory reactions. Among several pathways leading up to the activation of NF-kB, the ligand binding to pro-inflammatory receptors of interleukin (IL)-1 receptor superfamily is the most important pathway. One member of the IL-1 receptor family is the toll-like receptor 4 (TLR4). The TLR4 serves as a binding partner to lipopolysaccharide (LPS) as well as to the molecules categorized as damage-associated molecular pattern (DAMP). High mobility group box-1 (HMGB1) is one such DAMP protein which is released from necrotic cells and binds to the TLR4 to activate TLR4/NF-kB axis [8].

The objective of this study was to evaluate the effects of a novel NF-kB inhibitor, EF24, on the inflammatory injury of liver in a rat model of HS. Chemically, EF24 is a chalcone compound, 3,5-bis(2-fluorobenzylidene)piperidin-4-one, which was synthesized as a curcumin analog for its more potent anti-proliferative activity than curcumin [9]. In this respect, EF24 has been investigated as an anti-cancer agent in both in vitro and in vivo models of cancer [10-12]. It has also been shown to potently suppress NF-kB activation, modulate phenotype, and reduces secretion of TNF-α and IL-6 in lipopolysaccharide-stimulated dendritic cell model of sterile inflammation [13]. The putative mechanism of its activity is based on its ability to inhibit the catalytic activity of IkappaB kinase β (IKKβ) [14]. IKKβ phosphorylates the inhibitor of NF-kBα (IkBα) and destines it towards proteolytic degradation. The degradation of IkBα liberates NF-kB for phosphorylation-dependent nuclear translocation and transcriptional activity. We have recently shown that EF24 treatment in a rat model of 50% HS reduces pulmonary inflammation, restores intestinal barrier function, and improves overall survival rate [15-17]. Here, we hypothesized that EF24 administration will reduce the activation of pro-inflammatory TLR4/NF-kB/COX-2 pathway and improve markers of liver function.

## Methods

EF24 was synthesized in-house by the procedure published elsewhere [12]. For all the experiments, a sterile solution of EF24 was prepared in water having endotoxin content less than 0.1 EU/ml (Caisson Laboratories, North Logan, UT). The primary rabbit antibodies against rat antigens were obtained from Cell Signaling Technology (CST, Danvers, MA) and Santa Cruz Biotechnology (SCBT, Santa Cruz, CA). Horseradish peroxidase (HRP)-conjugated secondary antibodies were from Sigma-Aldrich (St. Louis, MO). All other chemicals were obtained from various vendors represented by VWR International (Radnor, PA).

### Rat model of fixed-volume hemorrhage

The animal experiments were performed according to the NIH Animal Use and Care Guidelines and were approved by the Institutional Animal Care and Use Committee of the University of Oklahoma Health Sciences Center. Male Sprague Dawley rats (250-300 g) were obtained from the breeding colony of Harlan Laboratories (Indianapolis, IN, USA). The rats were housed in regular light/dark cycles of 12/12. Before initiating the experiment, the rats were allowed to acclimatize for at least 5 days. An arterial catheter was implanted in the left femoral artery in an aseptic manner. The catheter was subcutaneously tunneled and secured to the nape. The rats were under isoflurane (3%) anesthesia in 100% oxygen stream (2 L per min) during the surgical procedure. The detailed method for femoral artery cannulation is described elsewhere [18]. The rats were allowed 2 days to recover from the cannulation surgery before their recruitment in the experiments. The cannulated rats were clustered in three groups: control (Ctrl, n = 6), HS only (HS, n = 6), and HS treated with EF24 (HS → EF24, n = 6). On the day of the experiment, the rats were handled under isoflurane (3%) anesthesia in a stream of medical grade air containing 21% oxygen (2 L per min). No attempt was made to regulate the core body temperature and the rats were allowed to freely access water and food. For uninterrupted blood withdrawal, the rats were heparinized with 100 units of heparin and HS was induced by withdrawing approximately 50% of circulating blood at the rate of 1.0 ml/min. The blood withdrawal was stopped when the desired volume of blood loss was reached, and the rats were allowed to wake up. The total volume of blood was estimated as 6% of bodyweight. After allowing the hypovolemic rats to freely compensate for 1 h, 0.4 mg/Kg bodyweight of EF24 in 100 μl isotonic volume was administered intraperitoneally. The rats in HS group received equal volume of vehicle in which EF24 was dissolved. The mean arterial blood pressure (MAP) and hematocrit were monitored at baseline, after hemorrhage, and before euthanasia as described previously [16]. The rats were euthanized after 5 h of EF24 administration to harvest liver tissue. The blood samples were collected upon termination of the study; plasma was separated for various assays.

### Growth-regulated gene product/cytokine-induced neutrophil chemoattractant (GRO/CINC-1) assay

GRO/CINC-1 is a rat analog of IL-8, which is produced by injured tissues and serves as a chemoattractant for neutrophils. We estimated GRO/CINC-1 in rat plasma collected at 6 h by using an immunoassay kit (Enzo Life Sciences, Plymouth Meeting, PA) and following the instructions provided with the kit.

### Myeloperoxidase (MPO) assay

The liver tissue-associated MPO activity is an indicator of neutrophil infiltration in inflammation. We performed assay in Ctrl, HS, and HS → EF24 groups. We used a colorimetric assay using O-dianisidine dihydrochloride as a peroxidase probe [17].

### Immunohistochemistry (IHC) and histopathology

We determined the expression levels of the TLR4, phospho-NF-κB p65, and COX-2 to assess the activation of pro-inflammatory pathway. The presence of these proteins in liver tissues was assessed by using an IHC kit from DakoCytomation (Carpinteria, CA). The sectioning and slide preparation of paraformaldehyde-fixed and paraffin-embedded tissues was performed by the core imaging facility of the Oklahoma Medical Research Foundation (OMRF, Oklahoma City, OK). For staining, the slides were blocked with a protein-block solution and incubated overnight with phospho-NF-κB-p65 (CST), COX-2 (SCBT), and the TLR4 (CST) antibodies at the dilutions of 1:200, 1:100, and 1:200, respectively. After functionalization with biotinylated link universal antiserum and a reaction with HRP-streptavidin conjugate, the color was developed using 3,3-diaminobenzidine hydrochloride. The sections were counterstained with Mayer’s hematoxylin solution. Digital brightfield images of immunostained slides were captured using Olympus IX-701 inverted microscope with an X40 objective lens and a DP70 camera (Olympus; Melville, NY). Three animals per treatment group were analyzed for each biomarker. Manual selection technique was used to classify the areas positively stained with DAB chromogen and counted directly on the screen [19,20]. For histopathologic examination, the paraffin-embedded tissues were stained with hematoxylin and eosin. The slides were examined by a board-certified veterinary pathologist for the assessment of liver injury, inflammation, and ischemic coagulative necrosis.

### Immunoblotting

The expression of the TLR4, phospho-NF-κB-p65, and COX-2 was also examined by immunoblotting. Since the transcriptional activity of NF-κB is regulated by phosphorylation of p65 subunit at Ser536 [21], the tissue expression of phospho-p65 subunit was taken as an indicator of NF-kB activation. The liver tissue homogenates were prepared in ice-cold buffer consisting of 10% Nonidet P-40, 5 M NaCl, 1 M HEPES, 0.1 M ethylene glycol tetraacetic acid, 0.5 M ethylenediaminetetraacetic acid, 1 M NaF, 0.2 M sodium orthovanadate, 0.1 M phenylmethylsulfonyl fluoride, 2 mg/ml aprotinin, and 2 mg/ml leupeptin. The proteins were fractionated by SDS-polyacrylamide gel electrophoresis, electrotransferred to the nitrocellulose membranes, blotted with primary antibodies against phospho-NF-κB-p65 (CST), the TLR4 (CST), HMGB1 (CST), and COX-2 (SCBT), followed by HRP-conjugated secondary antibody. The immunoreactive bands were detected by SuperSignal West Femto detection reagent (Thermo Fisher Scientific, Rockford, IL). To ensure equal protein loading, the membranes were stripped using a stripping solution containing 10% SDS and 0.5 M Tris and β-mercaptoethanol at 60°C for 45 minutes, and reprobed with anti-actin antibody (Sigma-Aldrich, St. Louis, MO). The membrane blocking reagents and the antibody dilutions were prepared according to the manufacturer’s recommendations.

### Systemic markers of liver injury

The plasma levels of aspartate transaminase (AST) and alanine transaminase (ALT) are clinical markers of hepatic injury. Likewise, the plasma levels of CD163 are indicative of the activation status of liver Kupffer cells [22,23]. The plasma levels of the AST and ALT were measured by using a kit from Biovision (Milpitas, CA). The plasma concentration of rat CD163 (also known as scavenger receptor cysteine-rich type 1 protein M130) was estimated by using an enzyme-linked immunoassay (ELISA) kit from MyBioSource (San Diego, CA).

### Apoptosis

The induction of apoptosis in liver was studied by immunoblotting (described above) for caspase-3, caspase-9, caspase-8, BAX, Bcl-2, and survivin. Caspase-9, caspase-8, caspase-3, and BAX are the pro-apoptotic proteins, whereas Bcl-2 and survivin are regarded as the anti-apoptotic proteins. The primary antibodies were acquired from CST. The fragmented DNA of apoptotic cells in liver tissue slices were end-labeled using a modified terminal deoxynucleotidyl transferase dUTP nick end labeling (TUNEL) assay supplied as a fluorometric kit (Promega, Madison, WI). The sectioning and staining of the tissues was performed in the OMRF’s core imaging facility (Oklahoma City, OK).

### Data analysis

The results were analyzed by analysis of variance (ANOVA) and the Bonferroni post-test using Prism software (GraphPad Software, Inc., San Diego, CA). The p value < 0.05 was considered statistically significant. The densitometry of immunoreactive bands was performed on at least three replicates for each experiment using ImageJ 1.46r freeware (National Institutes of Health).

## Results

We describe the effects of EF24 administration on hepatic TLR4/NF-kB/COX-2 pathway of inflammation, markers of liver injury (tissue MPO and HMGB1 expression; plasma AST and ALT), apoptosis (caspases, BAX, Bcl-2, and survivin), and the plasma levels of CD163 as an indicator of macrophage activation in rats with 50% HS. After blood loss, the mean arterial blood pressure in this model dropped from 97.5 ± 6.9 mm Hg at baseline (hematocrit = 44.1 ± 1.3) to 43 ± 4.1 mm Hg after hemorrhage (hematocrit = 27.4 ± 1.1).

### EF24 ameliorates HS-induced liver injury

**Figure 1:**
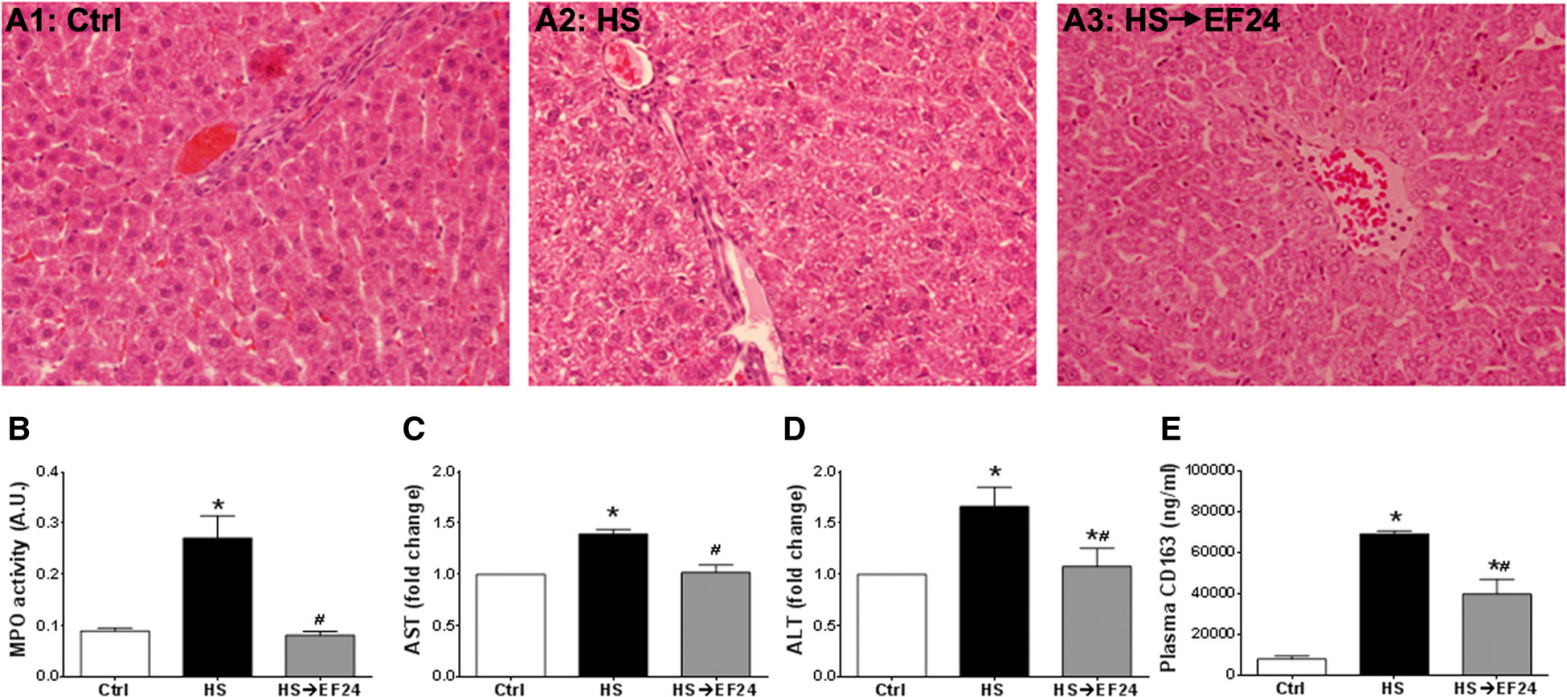
**Effects of hemorrhagic shock and EF24 treatment on liver. (A1-A3)** Histopathology of liver tissues from a representative set of rats, showing EF24-assisted recovery from hemorrhage-induced damages. The tissue slides were stained with hematoxylin & eosin and digital micrographs were obtained at 100X magnification. **(B)** Myeloperoxidase (MPO) activity in liver tissues (n = 5/group). **(C)** AST (n = 6/group) and **(D)** ALT (n = 6/group) levels in plasma collected at 6 h after HS. **(E)** Plasma levels of CD163 (n = 5/group) at 6 h after HS. * p < 0.05 vs. Ctrl and # p < 0.05 vs. HS.

The acute histopathologic consequences of HS in liver are shown in Figure 1A. At necropsy, the effect of blood loss was blanching of the liver lobes which remained so even after EF24 treatment, but no apparent areas of macroscopic necrosis were observed. There was also no histologic evidence of ischemic necrosis that would have first developed within the centrilobular zones. Under the microscope, the control liver slices revealed normal architecture, with a preserved trabecular structure of parenchyma cells. In rats suffering from HS, the hepatocellular architecture was deranged, characterized by mild to moderate cytoplasmic swelling with partial loss of the sinusoidal spaces in hemorrhaged rats. These changes were substantially reduced in liver of rats treated with EF24 (0.4 mg/Kg).

Treatment with EF24 markedly ameliorated the HS-induced increase in MPO activity, as well as the plasma levels of AST, ALT and CD163 (Figure 1B-E). As shown in Figure 1B, HS increased the tissue-associated MPO activity by 3-folds (Ctrl 0.09 ± 0.005 vs. HS 0.27 ± 0.043, p < 0.05) and treatment with EF24 reduced it to 0.08 ± 0.007 (p < 0.05, HS→EF24 vs. HS). Similarly, HS increased the plasma AST and ALT levels by approximately 40% and 66% over control levels, respectively (Ctrl vs. HS, p < 0.05), which were normalized to normal levels by EF24 treatment (p < 0.05, HS→EF24 vs. HS) (Figure 1C and D). The HS-induced increase in CD163 concentration in plasma was more than 8-folds, from control levels of 8.1 ± 1.4 to 68.9 ± 1.6 μg/ml in HS (Ctrl vs. HS, p < 0.05) (Figure 1E). EF24 treatment reduced the soluble CD163 in plasma to 39.8 ± 7.0 μg/ml (p <0.05, HS→EF24 vs. HS), but these circulating levels were significantly above the control levels (p < 0.05, HS→EF24 vs. Ctrl).

**Figure 2:**
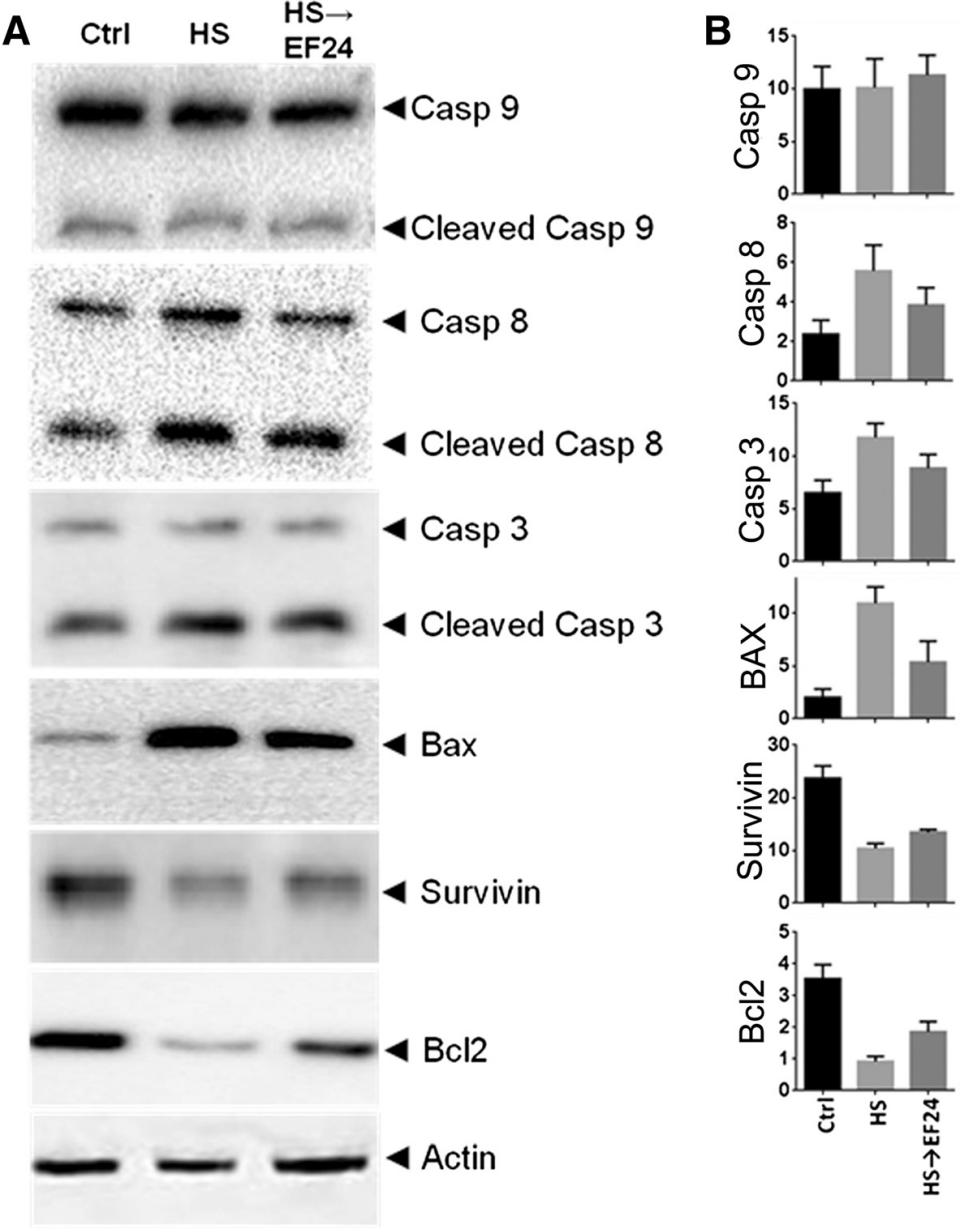
**Effects of hemorrhagic shock and EF24 treatment on apoptotic markers. (A)** Expression of caspase-3, caspase-9, caspase-8, Bcl-2, Bax, and survivin in liver tissues (n = 3/group). **(B)** The membranes were stripped and re-probed with anti-β-actin antibody to determine the actin-normalized densitometry units. Sham surgery (Ctrl), hemorrhage (HS) and hemorrhage with EF24 administration (HS → EF24).

Figure 2 shows that HS induced the expression of BAX and mild cleavage of caspase-8 and caspase-3, but there was no cleavage of caspase-9. Simultaneously, there was a reduction in the expression of Bcl-2 and survivin. When the hemorrhaged rats were treated with EF24, the expression levels of these proteins tended to shift back to their basal levels, suggesting that EF24 treatment blocked the weak induction of extrinsic apoptotic pathway. None of the changes in HS and EF24-treated groups satisfied the statistical significance of p < 0.05. The absence of marked induction of apoptosis was also supported by the lack of clear evidence in fluorescence microscopy of TUNEL-stained liver slices. The TUNEL-positivity among liver cells from various groups of rats was not different (data not shown).

### EF24 suppresses HS-induced CINC-1 expression in liver

**Figure 3:**
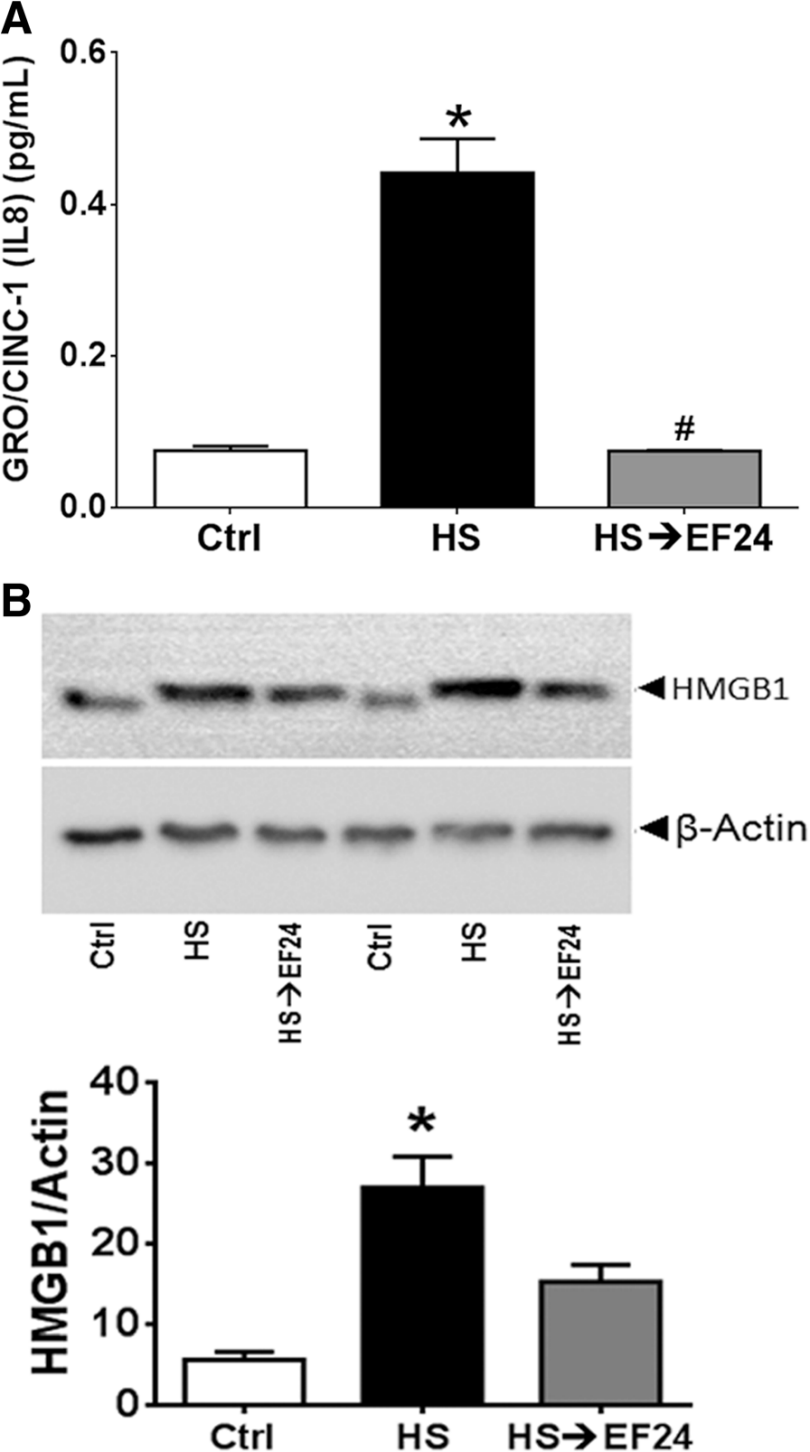
**EF24 treatment reduces hemorrhage-induced CINC-1 and HMGB1 expression. (A)** CINC expression in liver tissue estimated by ELISA (n = 4/group). **(B)** HMGB1 expression in liver tissue of hemorrhaged rats treated with EF24 (n = 4/group). The protein expression of HMGB1 was observed by immunoblotting (Upper panel), and the actin-normalized densitometry units were calculated (Lower panel). * p < 0.05 vs. Ctrl and # p < 0.05 vs. HS.

Figure 3A shows that HS caused a significant increase in liver tissue-associated CINC-1 from 0.08 ± 0.006 to 0.44 ± 0.044 pg/ml (Ctrl vs. HS, p < 0.05). EF24 treatment significantly lowered the HS-induced hepatic CINC-1 levels to 0.075 pg/ml (p < 0.05, HS→EF24 vs. HS). In addition, by immunoblotting we found that HS induced the expression of HMGB1 by approximately 5-folds (Ctrl vs. HS, p < 0.05), which was substantially reduced by EF24 treatment (Figure 3B). Interestingly, the mobility of HMGB1 band in hemorrhaged liver was conspicuously slowed as compared to that observed in case of tissues from control and EF24-treated rats.

### EF24 inhibits HS-induced TLR4 expression, NF-κB activation, and COX-2 expression

**Figure 4:**
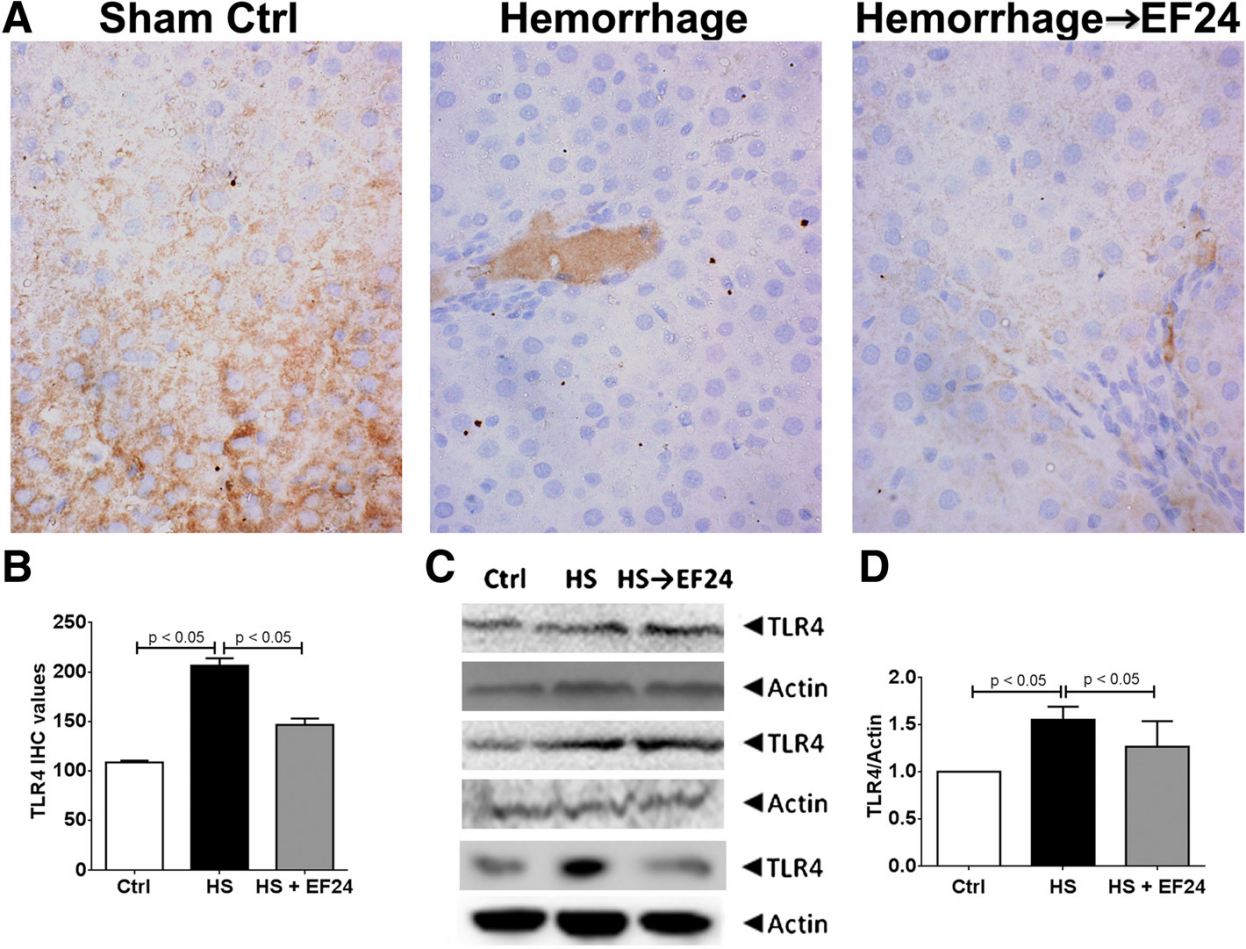
**EF24 reduces hemorrhage-induced expression of TLR4. (A)** Immunohistochemical analysis of TLR4 expression in liver tissues of rats subjected to sham surgery (Ctrl), hemorrhage (HS) and hemorrhage with EF24 administration (HS → EF24). The light blue stain represents the degree of expression of TLR4. **(B)** Quantitation of IHC values (* p < 0.05 vs. Ctrl and # p < 0.05 vs. HS; n = 3/group). **(C)** Immunoblots showing the TLR4 protein expression in three different sets. To demonstrate equal loading, the membranes were stripped and re-probed with anti-β-actin antibody. **(D)** Actin-normalized densitometry of TLR4 immunoblots.

The IHC staining of liver slices demonstrated that HS significantly increased the expression levels of the TLR4 (Figure 4A and B). The TLR4 positive cells increased from 109 ± 1.8 (mean ± sem) in control groups to 206 ± 7.5 in HS group (p < 0.05). EF24 treatment reduced the TLR4 positivity to 147 ± 6.4 (p < 0.05, vs. HS). The immunoblots for the TLR4 expression and the actin-normalized densitometry of blots also provided evidence of changes in the TLR4 expression after HS and EF24 treatment (Figure 4C and D). The expression of the TLR4 was increased in HS group by about 1.6 times and EF24 treatment brought it down to approximately 1.26 times as compared to the control levels. Whereas the HS-induced change in the TLR4 expression was significant (p <0.05, vs. Ctrl), the reduction caused by EF24 treatment was not statistically significant with respect to densitometry.

**Figure 5:**
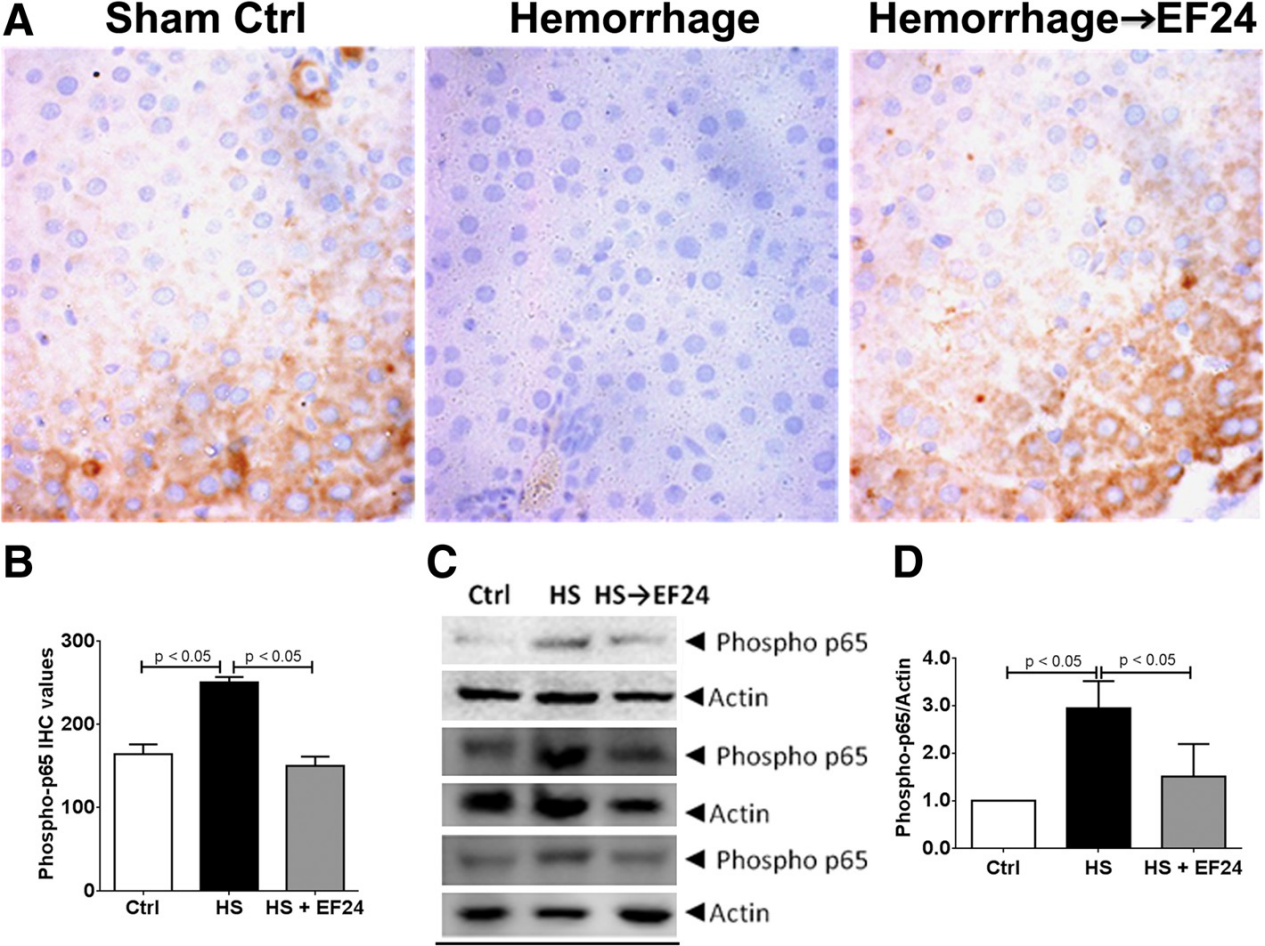
**EF24 reduces hemorrhage-induced expression of phospho-NF-κB p65. (A)** Immunohistochemical analysis of phospho-NF-kB p65 expression in liver tissues of rats subjected to sham surgery (Ctrl), hemorrhage (HS) and hemorrhage with EF24 administration (HS → EF24). The light blue stain represents the degree of expression of phospho-p65. **(B)** Quantitation of IHC values (* p < 0.05 vs. Ctrl and # p < 0.05 vs. HS; n = 3/group). **(C)** Immunoblots showing the phospho-NF-kB p65 expression in three different sets. To demonstrate equal loading, the membranes were stripped and re-probed with anti-β-actin antibody. **(D)** Actin-normalized densitometry of the immunoblots.

The IHC examination also revealed a clear induction of the phospho-NF-κB p65 expression in liver tissue after HS. The treatment with EF24 prevented this induction in a significant manner (Figure 5A and B). The stain positivity for the phospho-NF-κB p65 expression in Ctrl, HS, and HS→EF24 groups was 164 ± 11.9, 251 ± 6.5, and 150 ± 10.9, respectively (p <0.05, Ctrl vs. HS and HS vs. HS→EF24). Furthermore, the immunoblotting of liver tissue homogenates for the phospho-NF-κB p65 expression confirmed that HS induced the expression of phospho-NF-κB p65 and EF24 treatment reduced this induction (Figure 5C and D). The densitometry-based analyses revealed that compared to the control group, the expression of phospho-NF-κB p65 increased by approximately 3 times in HS group (p < 0.05, vs. Ctrl), whereas EF24 treatment reduced the expression to approximately 1.5 times (p < 0.05, vs. HS).

**Figure 6:**
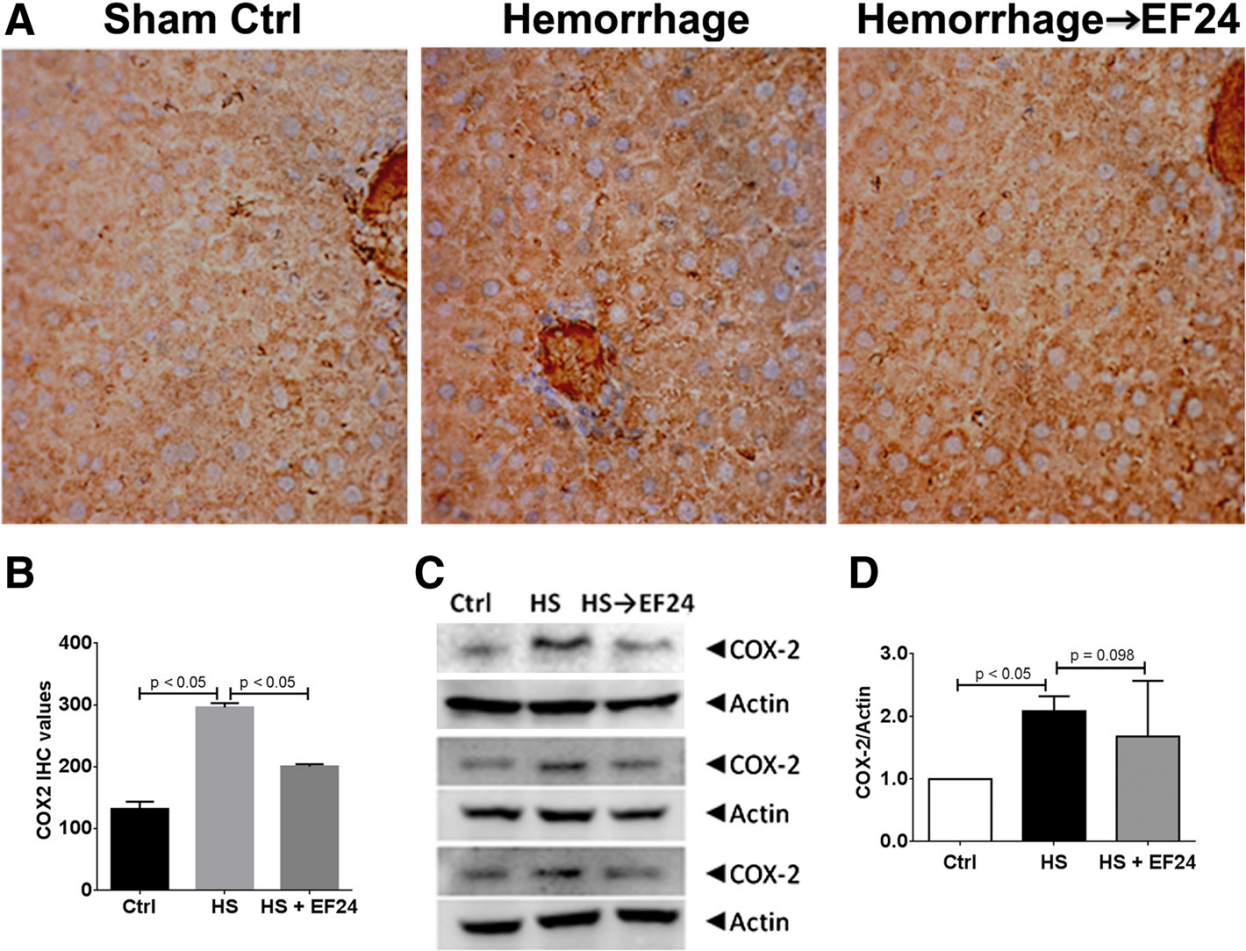
**EF24 reduces hemorrhage-induced expression of COX-2. (A)** Immunohistochemical analysis of COX-2 expression in liver tissues of rats subjected to sham surgery (Ctrl), hemorrhage (HS) and hemorrhage with EF24 administration (HS → EF24). The light blue stain represents the degree of expression of COX-2. **(B)** Quantitation of IHC values (* p < 0.05 vs. Ctrl and # p < 0.05 vs. HS; n = 3/group). **(C)** Immunoblots showing COX-2 expression in three different sets. To demonstrate equal loading, the membranes were stripped and re-probed with anti-β-actin antibody. **(D)** Actin-normalized densitometry of COX-2 immunoblots.

Like the TLR4 and the phospho-NF-κB p65 expression, the IHC (Figure 6A and B) and immunoblotting (Figure 6C and D) evidence for COX-2 expression also showed that EF24 treatment inhibited the HS-induced COX-2 expression in liver tissue. The number of COX-2 positive cells increased from control levels of 132 ± 11.6 to 297 ± 6.9 in HS group (p < 0.05, vs. Ctrl). The number of COX-2 positive cells reduced to approximately 201 ± 3.7 in EF24-treated rat liver (p < 0.05, vs. HS). Based on the densitometry of immunoblots, in comparison to the control level, the expression levels of COX-2 in HS and HS→EF24 groups was 2.09 ± 0.24 and 1.68 ± 0.29, respectively (p <0.05, Ctrl vs. HS and p = 0.098, HS vs. HS→EF24).

## Discussion

We previously reported the salutary effects of EF24 on lungs of rats with HS [15,16]. EF24 treatment increased the 6 h survival in this model from 36% in the untreated group to 75% in the treated group of rats [16]. The liver is another organ of immense importance in HS. HS not only diminishes volume-flow through the hepatic artery and portal vein, but also reduces oxygen saturation of the venous blood from 15% to ≤ 5% in the portal vein which accounts for approximately 75% of blood supply to the liver [24]. The resultant hypoperfusion and hypoxia often results in irreversible hepatic injury if the low flow state is allowed to persist for long, or the hemodynamics-preserving resuscitation is delayed [25]. Liver failure in shock is a part of the multiple organ dysfunction syndrome, the principal driver of which is the excessive activation of inflammatory pathways in the affected organs [26,27]. It has been suggested that modulation of inflammatory process by inhibiting the TLR4/NF-kB axis could be exploited for mitigation of the organ damage caused by HS [28,29]. In this regard, EF24 has been shown to inhibit NF-kB activation by interfering with the catalytic activity of IkappaB kinase β (IKKβ) [14]. Chemically, EF24 belongs to the chalcone group of compounds. Chalcones are mostly researched for their anti-cancer activity [30-32], but none has been investigated for the mitigation of SIRS in shock. Herein, we describe the salutary effects of EF24 administration on HS-associated liver damage.

NF-kB is an important pivot in signaling of the IL-1R superfamily which includes the TLR4. The TLR4-ligand interaction could activate the NF-κB-dependent as well as NF-κB-independent inflammatory pathways. Although the TLR4 is placed upstream of NF-kB pathway, our results indicate that EF24 also reduces the expression of the TLR4. This could be because of the transcriptional control exercised by NF-kB over the TLR4 expression. NF-κB recruits E2F1 as a transcription partner to activate its genome-wide LPS-responsive genes including the TLR4 [33]. NF-κB also transcriptionally controls the *COX-2* gene that is inducible by HS and reperfusion injury. The eicosanoids generated from COX-2 activation are important players in the manifestation of inflammatory pathology. We found that EF24 treatment subdued the induction of COX-2 protein in hemorrhaged liver tissue. In addition to the eicosanoids generated from COX-2 induction, the effector soluble factors in the NF-kB pathway also include the cytokines, such as TNF-α and IL-6; a strong correlation between the serum cytokines levels and the therapeutic outcome exists in victims of shock [34]. EF24 was found to significantly suppress the expression of CINC-1 in liver tissue (Figure 3). CINC-1 is a powerful neutrophil chemoattractant expressed by macrophages in the inflamed tissue. A direct link also exist between the suppression of pro-inflammatory TLR4/NF-kB axis by EF24 treatment and the reduced CD163 expression and shedding. Shedding of CD163 into the circulation has been shown to occur after the TLR activation [35]. The expression of CD163 antigen in the macrophages itself has been shown to be controlled by various NF-kB-regulated inflammatory mediators such as interferon γ and IL-6, and it is thought that CD163-expressing cells have a role in regulation of the immune response [36,37]. Furthermore, COX-generated prostaglandin E2 has been reported to play a direct role in regulating the expression of CD163 on monocytes in human liver tissues [38]. Thus, it could be hypothesized that the reduced levels of plasma CD163 in EF24-treated rats is a result of the suppression of inflammatory signaling in the ischemic and hypo-perfused liver.

Cell death in the liver occurs mainly by apoptosis or necrosis, with participation of other variants (necroptosis, autophagy, and pyroptosis) depending upon the nature and duration of insult [39]. The biochemical evidence for apoptotic cell death in our model of HS was not convincing (Figure 2) because there was no induction of caspase-9, weak activation of caspase-8 and caspase-3, and negative TUNEL staining in the liver tissue. The only sure signs related to apoptosis were the HS-induced decreased expression of anti-apoptotic Bcl-2 and increased expression of pro-apoptotic Bax protein. However, Bcl-2 and Bax expression levels are not the conclusive proofs of apoptosis because they also participate in necrotic cell death [40,41]. The lack of apoptotic signatures in our model is different from the observations made in several other investigations, perhaps because the other studies administered resuscitation fluids in their study models [42-44]. In contrast, we employed a model of prolonged ischemia without any fluid resuscitation. It has been postulated that apoptotic cell death occurs mainly during the restoration of the circulation and oxygen supply, whereas ischemia associated with massive blood loss causes hepatocytes to undergo necrosis [45]. Presumably, the energy needs associated with the apoptosis process restrict it to occur only during the reperfusion phase because it depends on oxidative metabolism in the cells [46]. Elsewhere, Paxian et al have reported that prolonged period of hemorrhagic hypotension was associated with necrosis whereas resuscitation within a short duration of shock induced apoptosis in hepatic tissue [47]. Therefore, we speculate that liver cells undergo type III or necrotic cell death after prolonged HS. However, the light microscopic pathology could not adequately define necrosis in our rat model. The mild hepatic injury observed in our rat model may be because of the short duration (6 h) of shock which perhaps is not sufficiently long for large scale histologic and functional changes to become evident.

The usual criteria employed to establish necrotic cell death are the absence of apoptosis and the release of intracellular proteins such as HMGB1 and cytokeratin 18 [48]. HMGB1 is a chromatin structural protein which is released in the extracellular milieu from trauma-afflicted necrotic cells, but remains bound to DNA in apoptotic cells. Upon extracellular release, HMGB1 acts like a DAMP and activates inflammatory pathways by interacting with the TLR4 and other pro-inflammatory receptors [8]. Given that EF24 treatment suppressed the expression of HMGB1 in the liver tissue of hemorrhaged rats, it appears that EF24 reduced the induction of necrotic cell death. Another interesting observation was the conspicuous mobility shift of the HMGB1 band in the immunoblots (Figure 4). We speculate that this mobility-shift is because of its altered state of acetylation. Since acetylation neutralizes the positive charge of the ε-amino group of lysine, there is an increase of the net negative charge of the SDS-protein complexes, without significantly changing the molecular mass of the protein [49]. Translocation of HMGB1 from nucleus to cytosol is favored by its hyperacetylation [50]. It has also been shown that the suppression of histone deacetylase activity in the nucleus contributes to the increase in acetylated HMGB1 release after oxidative stress in ischemic hepatocytes [51].

The plasma levels of liver transaminases ALT and AST are reliable markers of hepatocellular injury and even mildly elevated levels of these enzymes are considered a sign of serious underlying condition. Likewise, plasma levels of CD163 are indicative of the activation status of the liver Kupffer cells [22,23]. The effect of EF24 treatment on the plasma AST and ALT levels in hemorrhaged rats suggested an improvement in the functional status of hepatic parenchyma. However, we speculate that the primary effect of EF24 is on the neighboring Kupffer cells because EF24 treatment significantly reduced the circulating levels of CD163 in HS (Figure 1E). CD163 is a macrophage lineage-specific endocytic scavenger receptor for haptoglobin-hemoglobin complexes which is a marker for activated macrophages [22,52]. The soluble form of CD163 (sCD163) has been shown to increase in plasma after liver injury [23]. Since Kupffer cells constitute the large majority of body macrophages, they are regarded as the primary source of sCD163 in circulation [22]. Secondly, the cytokines, eicosanoids, oxygen free radicals and enzymes released by activated Kupffer cells influence the metabolic activity of the liver parenchymal cells [53]. A paracrine circuit between the activated Kupffer cells and hepatocytes is an early event in the induction of post-ischemic oxidative stress in the liver [54].

## Conclusions

In summary, we demonstrate for the first time that an NF-kB inhibitor EF24 has a potent salutary effect on liver of severely hemorrhaged rats. By inhibiting the pro-inflammatory TLR4/NF-kB/COX-2 pathway signaling, EF24 improved the classical serum biomarkers of hepatic injury. The encouraging results of this study warrant further trials involving EF24 in combination with crystalloids or whole blood resuscitation in a reperfusion model. Furthermore, it will be of interest to investigate the target cell-type for EF24 activity. Based on our previous work in lipopolysaccharide-stimulated dendritic cells and EF24 [13], we hypothesize that the anti-inflammatory effect of EF24 is primarily on the resident macrophages and its salutary effect on hepatocytic function may be secondary. This hypothesis is also supported by our observations about the circulating levels of CD163 as described above.

## References

[1] Heckbert SR, Vedder NB, Hoffman W, Winn RK, Hudson LD, Jurkovich GJ (1998). Outcome after hemorrhagic shock in trauma patients. J Trauma.

[2] Helling TS (2005). The liver and hemorrhagic shock. J Am Coll Surg.

[3] Kobelt F, Schreck U, Henrich HA (1994). Involvement of liver in the decompensation of hemorrhagic shock. Shock.

[4] Hsu JT, Kuo CJ, Chen TH, Wang F, Lin CJ, Yeh TS (2012). Melatonin prevents hemorrhagic shock-induced liver injury in rats through an Akt-dependent HO-1 pathway. J Pineal Res.

[5] Kao KK, Fink MP (2010). The biochemical basis for the anti-inflammatory and cytoprotective actions of ethyl pyruvate and related compounds. Biochem Pharmacol.

[6] Hierholzer C, Billiar TR (2001). Molecular mechanisms in the early phase of hemorrhagic shock. Langenbecks Arch Surg.

[7] Guillemin K, Krasnow MA (1997). The hypoxic response: huffing and HIFing. Cell.

[8] Andersson U, Tracey KJ (2011). HMGB1 is a therapeutic target for sterile inflammation and infection. Annu Rev Immunol.

[9] Adams BK, Ferstl EM, Davis MC, Herold M, Kurtkaya S, Camalier RF (2004). Synthesis and biological evaluation of novel curcumin analogs as anti-cancer and anti-angiogenesis agents. Bioorg Med Chem.

[10] Subramaniam D, May R, Sureban SM, Lee KB, George R, Kuppusamy P (2008). Diphenyl difluoroketone: a curcumin derivative with potent in vivo anticancer activity. Cancer Res.

[11] Agashe H, Lagisetty P, Sahoo K, Bourne D, Grady B, Awasthi V (2011). Liposome-encapsulated EF24-HPβCD inclusion complex: A preformulation study and biodistribution in a rat model. J Nanoparticle Res..

[12] Lagisetty P, Powell DR, Awasthi V (2009). Synthesis and structural determination of 3 3,5-bis(2-fluorobenzylidene)-4-piperidone analogs of curcumin. J Mol Str.

[13] Vilekar P, Awasthi S, Natarajan A, Anant S, Awasthi V (2012). EF24 suppresses maturation and inflammatory response in dendritic cells. Int Immunol.

[14] Kasinski AL, Du Y, Thomas SL, Zhao J, Sun SY, Khuri FR (2008). Inhibition of IkappaB kinase-nuclear factor-kappaB signaling pathway by 3,5-bis(2-flurobenzylidene)piperidin-4-one (EF24), a novel monoketone analog of curcumin. Mol Pharmacol.

[15] Yadav VR, Vilekar P, Awasthi S, Awasthi V. Hemorrhage-induced interleukin-1 receptor pathway in lung is suppressed by 3,5-bis(2-fluorobenzylidine)-4-piperidine (EF24) in a rat model of hypovolemic shock. Artificial Organs. 2014; In press. 10.1111/aor.12305 PMC4146623 24749913

[16] Yadav VR, Sahoo K, Roberts PR, Awasthi V (2013). Pharmacologic suppression of inflammation by a diphenyldifluoroketone, EF24, in a rat model of fixed-volume hemorrhage improves survival. J Pharmacol Exp Ther.

[17] Yadav VR, Hussain A, Sahoo K, Awasthi V (2014). Remediation of Hemorrhagic Shock-Induced Intestinal Barrier Dysfunction by Treatment with Diphenyldihaloketones EF24 and CLEFMA. J Pharmacol Exp Ther.

[18] Awasthi V, Yee SH, Jerabek P, Goins B, Phillips WT (2007). Cerebral oxygen delivery by liposome-encapsulated hemoglobin: a positron-emission tomographic evaluation in a rat model of hemorrhagic shock. J Appl Physiol.

[19] Walker RA (2006). Quantification of immunohistochemistry–issues concerning methods, utility and semiquantitative assessment I. Histopathology.

[20] Taylor CR, Levenson RM (2006). Quantification of immunohistochemistry–issues concerning methods, utility and semiquantitative assessment II. Histopathology.

[21] Sakurai H, Suzuki S, Kawasaki N, Nakano H, Okazaki T, Chino A (2003). Tumor necrosis factor-alpha-induced IKK phosphorylation of NF-kappaB p65 on serine 536 is mediated through the TRAF2, TRAF5, and TAK1 signaling pathway. J Biol Chem.

[22] Gronbaek H, Sandahl TD, Mortensen C, Vilstrup H, Moller HJ, Moller S (2012). Soluble CD163, a marker of Kupffer cell activation, is related to portal hypertension in patients with liver cirrhosis. Aliment Pharmacol Ther.

[23] Hiraoka A, Horiike N, Akbar SM, Michitaka K, Matsuyama T, Onji M (2005). Soluble CD163 in patients with liver diseases: very high levels of soluble CD163 in patients with fulminant hepatic failure. J Gastroenterol.

[24] Fine J, Seligman AM, Frank HA (1947). On the specific role of the liver in hemorrhagic shock: report of progress to date. Ann Surg.

[25] Matot I, Cohen K, Pappo O, Barash H, Abramovitch R (2008). Liver response to hemorrhagic shock and subsequent resuscitation: MRI analysis. Shock.

[26] Bone RC (1996). Immunologic dissonance: a continuing evolution in our understanding of the systemic inflammatory response syndrome (SIRS) and the multiple organ dysfunction syndrome (MODS). Ann Intern Med.

[27] Peitzman AB, Billiar TR, Harbrecht BG, Kelly E, Udekwu AO, Simmons RL (1995). Hemorrhagic shock. Curr Probl Surg.

[28] Doursout M-FJ, Liang YY, Uray KS, Pati S, Matijevic N, Holcomb JB. Role of the NF-kB pathway in rats subjected to moderate hemorrhage. FASEB J. 2009; 23 (Meeting Abstract Supplement):LB380.

[29] Prince JM, Levy RM, Yang R, Mollen KP, Fink MP, Vodovotz Y (2006). Toll-like receptor-4 signaling mediates hepatic injury and systemic inflammation in hemorrhagic shock. J Am Coll Surg.

[30] Yadav VR, Prasad S, Sung B, Aggarwal BB (2011). The role of chalcones in suppression of NF-kappaB-mediated inflammation and cancer. Int Immunopharmacol.

[31] Batovska DI, Todorova IT (2010). Trends in utilization of the pharmacological potential of chalcones. Curr Clin Pharmacol.

[32] Sahu NK, Balbhadra SS, Choudhary J, Kohli DV (2012). Exploring pharmacological significance of chalcone scaffold: a review. Curr Med Chem.

[33] Lim CA, Yao F, Wong JJ, George J, Xu H, Chiu KP (2007). Genome-wide mapping of RELA(p65) binding identifies E2F1 as a transcriptional activator recruited by NF-kappaB upon TLR4 activation. Mol Cell.

[34] Roumen RM, Hendriks T, van der Ven-Jongekrijg J, Nieuwenhuijzen GA, Sauerwein RW, van der Meer JW (1993). Cytokine patterns in patients after major vascular surgery, hemorrhagic shock, and severe blunt trauma. Relation with subsequent adult respiratory distress syndrome and multiple organ failure. Ann Surg.

[35] Weaver LK, Hintz-Goldstein KA, Pioli PA, Wardwell K, Qureshi N, Vogel SN (2006). Pivotal advance: activation of cell surface Toll-like receptors causes shedding of the hemoglobin scavenger receptor CD163. J Leukoc Biol.

[36] Hogger P, Dreier J, Droste A, Buck F, Sorg C (1998). Identification of the integral membrane protein RM3/1 on human monocytes as a glucocorticoid-inducible member of the scavenger receptor cysteine-rich family (CD163). J Immunol.

[37] Buechler C, Ritter M, Orso E, Langmann T, Klucken J, Schmitz G (2000). Regulation of scavenger receptor CD163 expression in human monocytes and macrophages by pro- and antiinflammatory stimuli. J Leukoc Biol.

[38] Zhang M, Ye Y, Wang F, Zhu J, Zhao Q, Zheng Y (2014). Liver myofibroblasts up-regulate monocyte CD163 expression via PGE2 during hepatitis B induced liver failure. J Transl Med.

[39] Guicciardi ME, Malhi H, Mott JL, Gores GJ (2013). Apoptosis and necrosis in the liver. Compr Physiol.

[40] Tsujimoto Y, Shimizu S, Eguchi Y, Kamiike W, Matsuda H (1997). Bcl-2 and Bcl-xL block apoptosis as well as necrosis: possible involvement of common mediators in apoptotic and necrotic signal transduction pathways. Leukemia.

[41] Whelan RS, Konstantinidis K, Wei AC, Chen Y, Reyna DE, Jha S (2012). Bax regulates primary necrosis through mitochondrial dynamics. Proc Natl Acad Sci U S A.

[42] Cheng Q, Yang G, Ma J, Li J, Shan Q (2014). Effects of different types of fluid resuscitation on hepatic mitochondria and apoptosis. Exp Ther Med.

[43] Jaskille A, Koustova E, Rhee P, Britten-Webb J, Chen H, Valeri CR (2006). Hepatic apoptosis after hemorrhagic shock in rats can be reduced through modifications of conventional Ringer's solution. J Am Coll Surg.

[44] Deb S, Martin B, Sun L, Ruff P, Burris D, Rich N (1999). Resuscitation with lactated Ringer's solution in rats with hemorrhagic shock induces immediate apoptosis. J Trauma.

[45] Karmaniolou II, Theodoraki KA, Orfanos NF, Kostopanagiotou GG, Smyrniotis VE, Mylonas AI (2013). Resuscitation after hemorrhagic shock: the effect on the liver–a review of experimental data. J Anesth.

[46] Leist M, Single B, Castoldi AF, Kuhnle S, Nicotera P (1997). Intracellular adenosine triphosphate (ATP) concentration: a switch in the decision between apoptosis and necrosis. J Exp Med.

[47] Paxian M, Bauer I, Rensing H, Jaeschke H, Mautes AE, Kolb SA (2003). Recovery of hepatocellular ATP and "pericentral apoptosis" after hemorrhage and resuscitation. FASEB J.

[48] Krysko DV, Vanden Berghe T, Parthoens E, D'Herde K, Vandenabeele P (2008). Methods for distinguishing apoptotic from necrotic cells and measuring their clearance. Methods Enzymol.

[49] Georgieva EI, Sendra R (1999). Mobility of acetylated histones in sodium dodecyl sulfate-polyacrylamide gel electrophoresis. Anal Biochem.

[50] Lu B, Antoine DJ, Kwan K, Lundback P, Wahamaa H, Schierbeck H (2014). JAK/STAT1 signaling promotes HMGB1 hyperacetylation and nuclear translocation. Proc Natl Acad Sci U S A.

[51] Evankovich J, Cho SW, Zhang R, Cardinal J, Dhupar R, Zhang L (2010). High mobility group box 1 release from hepatocytes during ischemia and reperfusion injury is mediated by decreased histone deacetylase activity. J Biol Chem.

[52] Moller HJ (2012). Soluble CD163. Scand J Clin Lab Invest.

[53] Strassburg CP (2003). Gastrointestinal disorders of the critically ill. Shock liver. Best Pract Res Clin Gastroenterol.

[54] Cutrin JC, Llesuy S, Boveris A (1998). Primary role of Kupffer cell-hepatocyte communication in the expression of oxidative stress in the post-ischaemic liver. Cell Biochem Funct.

